# P-1835. Evolution of Antibiotic Resistance Patterns in *Acinetobacter baumannii* Clinical Isolates in Response to Bleach Exposure

**DOI:** 10.1093/ofid/ofae631.1998

**Published:** 2025-01-29

**Authors:** Justin Halim, Valerie J Carabetta, Michael Curry, Nicholas Libraro, Rachel Carr

**Affiliations:** Cooper Medical School of Rowan University, Camden, New Jersey; Cooper Medical School of Rowan University, Camden, New Jersey; Cooper Medical School of Rowan University, Camden, New Jersey; Cooper Medical School of Rowan University, Camden, New Jersey; Cooper Medical School of Rowan University, Camden, New Jersey

## Abstract

**Background:**

*Acinetobacter baumannii* (Ab) is a common cause of drug-resistant infections. Ab rapidly acquires antibiotic resistance and can survive exposure to numerous antiseptics such as bleach. Prior research has demonstrated increased antibiotic resistance in Ab following exposure to antiseptics, likely due to increased expression of efflux pumps, but the relationship between antiseptic resistance and antibiotic resistance is not well known. In this study, we describe the evolution of antibiotic resistance in two Ab isolates following exposure to bleach.

**Table 1:**
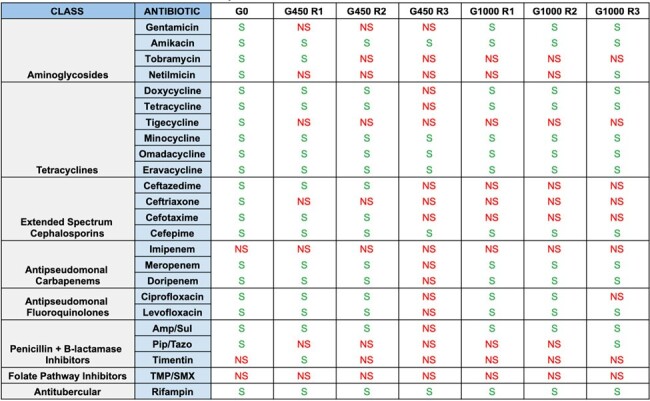
Antibiotic Susceptibilities for Evolved Strains of ACB3

Antibiotic susceptibilities for the evolved strains of ACB3 are depicted. For each strain, antibiotic susceptibilities were determined based on Clinical & Laboratory Standards Institute (CLSI) reference standards, and were classified as susceptible (S) or non-susceptible (NS). Non-susceptible values were those that were determined to be either intermediate or resistant. ACB3 was initially susceptible to most antibiotics at G0, and developed increased resistance to all antibiotic classes by G450.

**Methods:**

Two Ab clinical isolates, ACB3 and ACB9, were exposed to sublethal (0.01%) concentrations of bleach and grown in triplicate, with strains selected at baseline (G0), the 450^th^ generation (G450), and the 1000^th^ generation (G1000) for further analysis. Antibiotic susceptibility profiles for each strain were established by determining minimum inhibitory concentration (MIC) values against 24 standard-of-care antibiotics using broth microdilution assays. Whole genome sequencing (WGS) of each strain was performed to evaluate for the emergence of new alleles to affecting antibiotic resistance.

**Table 2:**
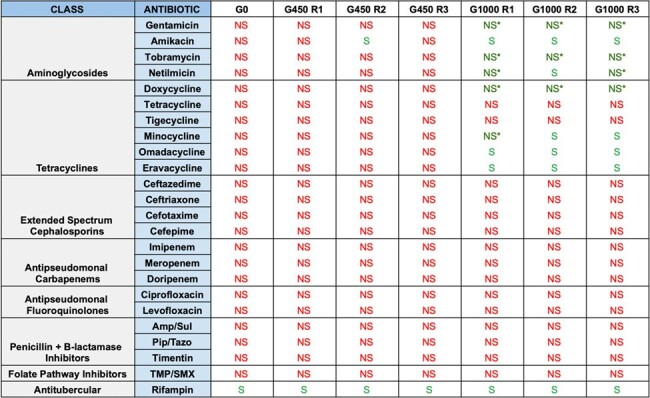
Antibiotic Susceptibilities for Evolved Strains of ACB9

Antibiotic susceptibilities for the evolved strains of ACB9 are depicted. For each strain, antibiotic susceptibilities were determined based on Clinical & Laboratory Standards Institute (CLSI) reference standards, and were classified as susceptible (S) or non-susceptible (NS). Non-susceptible values were those that were determined to be either intermediate or resistant. NS* denotes strains with MIC values that were decreased, but not enough to be classified as susceptible. ACB9 was initially PDR at G0 but developed increased susceptibility to all aminoglycoside and tetracycline drugs by G1000.

**Results:**

The ACB3 line was susceptible to most antibiotics at G0, but developed increased resistance to various antibiotics by G450, particularly against aminoglycoside and beta-lactam drugs (Table 1). Conversely, the ACB9 line was pan-drug resistant (PDR) at G0 but developed increased susceptibility to all aminoglycoside and tetracycline drugs by G1000 (Tables 2, 3). WGS of ACB9 G1000 demonstrated mutations in *uvrA* and *uvrB*, which encode nucleotide excision repair proteins, with concomitant mutations in the ABC transporter TssG, and type IV secretion system (T4SS) proteins TssC and VgrG. Mutations were observed in numerous other unidentified genes with close homology to ABC transporters, MFS efflux pumps, and OprD porins.

**Table 3:**
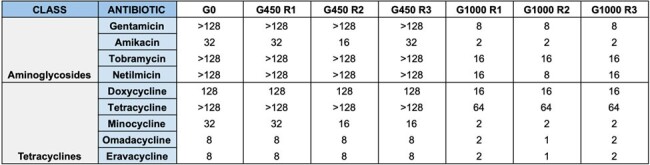
Minimum Inhibitor Concentration (MIC) Values for Aminoglycoside and Tetracycline Drugs Against Evolved Strains of ACB9

MIC values for aminoglycoside and tetracycline drugs against evolved strains of ACB9 are depicted. All three triplicates of ACB9 were initially resistant to all aminoglycoside and tetracycline drugs tested at G0. By G1000, all three triplicates demonstrated increased susceptibility to all aminoglycoside and tetracycline drugs, as indicated by decreased MIC values.

**Conclusion:**

We describe the evolution of antibiotic resistance in Ab after exposure to bleach. ACB3 was initially susceptible to most antibiotics but developed increased resistance. ACB9 was initially PDR but developed increased susceptibility to aminoglycoside and tetracycline drugs, likely due to mutations in hypermutators *uvrA* and *uvrB,* enabling mutations in genes which may confer resistance to these drugs.

**Disclosures:**

**All Authors**: No reported disclosures

